# Identification of Algerian Field-Caught Phlebotomine Sand Fly Vectors by MALDI-TOF MS

**DOI:** 10.1371/journal.pntd.0004351

**Published:** 2016-01-15

**Authors:** Ismail Lafri, Lionel Almeras, Idir Bitam, Aurelia Caputo, Amina Yssouf, Claire-Lise Forestier, Arezki Izri, Didier Raoult, Philippe Parola

**Affiliations:** 1 Aix Marseille Université, URMITE, UM63, CNRS 7278, IRD 198, Inserm 1095, Marseille, France; 2 Ecole Nationale Supérieure Vétérinaire d’Alger, Alger, Algérie; 3 Institut des Sciences Vétérinaires, Université Blida 1, Blida, Algérie; 4 Université de Bab Ezzouar, Laboratoire d’Ecologie et Environnement, Bab Ezzouar, Algérie; 5 Parasitologie-Mycologie, CHU Avicenne, Université Paris 13, Bobigny, France; Liverpool School of Tropical Medicine, UNITED KINGDOM

## Abstract

**Background:**

Phlebotomine sand flies are known to transmit *Leishmania* parasites, bacteria and viruses that affect humans and animals in many countries worldwide. Precise sand fly identification is essential to prevent phlebotomine-borne diseases. Over the past two decades, progress in matrix-assisted laser desorption/ionization time-of-flight mass spectrometry (MALDI-TOF MS) has emerged as an accurate tool for arthropod identification. The objective of the present study was to investigate the usefulness of MALDI-TOF MS as a tool for identifying field-caught phlebotomine.

**Methodology/Principal Findings:**

Sand flies were captured in four sites in north Algeria. A subset was morphologically and genetically identified. Six species were found in these areas and a total of 28 stored frozen specimens were used for the creation of the reference spectrum database. The relevance of this original method for sand fly identification was validated by two successive blind tests including the morphological identification of 80 new specimens which were stored at -80°C, and 292 unknown specimens, including engorged specimens, which were preserved under different conditions. Intra-species reproducibility and inter-species specificity of the protein profiles were obtained, allowing us to distinguish specimens at the gender level. Querying of the sand fly database using the MS spectra from the blind test groups revealed concordant results between morphological and MALDI-TOF MS identification. However, MS identification results were less efficient for specimens which were engorged or stored in alcohol. Identification of 362 phlebotomine sand flies, captured at four Algerian sites, by MALDI-TOF MS, revealed that the subgenus *Larroussius* was predominant at all the study sites, except for in M’sila where *P*. *(Phlebotomus) papatasi* was the only sand fly species detected.

**Conclusion:**

The present study highlights the application of MALDI-TOF MS for monitoring sand fly fauna captured in the field. The low cost, reliability and rapidity of MALDI-TOF MS analyses opens up new ways in the management of phlebotomine sand fly-borne diseases.

## Author Summary

Phlebotomine sand flies are known to transmit *Leishmania* parasites, bacteria and viruses that affect humans and animals in many countries worldwide. Algeria is among the top ten countries with the highest number of cutaneous leishmaniasis cases. To prevent the emergence of leishmaniasis and to establish vector control and monitoring, precise identification of sand fly specimens at the species level is essential. Current morphological and molecular methods are long, expensive and laborious, limiting large-scale surveys. The recent application of MALDI-TOF MS for the identification of arthropods, including sand flies, opens up new opportunities in this field. Here, for the first time, this proteomic tool was assessed for the identification of sand flies collected in the field from leishmaniasis-endemic Algerian areas. Six distinct sand fly species were found and a reference spectrum database was created. Correct identification was obtained for all non-engorged blind-tested specimens stored by freezing. As a result, this proteomic tool could be applied for the classification of phlebotomine sand fly specimens caught in the field, enabling entomological surveys to be conducted.

## Introduction

Phlebotomine sand flies (Diptera: Psychodidae) are small, blood-sucking insects that feed on a wide range of hosts, and potentially act as vectors for pathogens responsible for human and animal diseases worldwide. Of more than 800 sand fly species which have been described to date, approximately 10% are suspected or proven vectors of bacteria, arboviruses including sandfly fever Sicilian and Toscana viruses, and parasites such as *Leishmania* [1]. Phlebotomine sand flies are the only haematophagous insects proven to transmit *Leishmania* parasites through biting infected females that have previously fed on a *Leishmania*-infected mammal [1].

*Leishmania* parasites are the causative agents of leishmaniases, affecting mainly the poorest of the poor in developing countries [2]. Currently, 350 million people are considered to be at risk of contracting leishmaniasis, and two million new cases occur each year [2]. Leishmaniasis occurs in two clinical forms: cutaneous leishmaniasis (CL) and visceral leishmaniasis (VL) [2]. Maghreb is known to be one of the most endemic areas of leishmaniases where both visceral and cutaneous forms are reported [3].

In North Africa, CL is more widely distributed than VL, and incidence and distribution have increased since the 1980s [4]. Algeria is one of the ten countries with the highest number of CL cases [2].

With the growing importance of phlebotomine-borne diseases in medicine and veterinary medicine, species identification is the first step in entomological and epidemiological studies and misidentification can have a negative impact on public health. The species-level identification of phlebotomine sand flies relies on the detection of morphologic criteria including the pharynx, spermathecae and cibarium for females and genitalia for males [5]. Nonetheless, morphological identification requires taxonomic expertise and is time-consuming, requiring each specimen’s discriminating criteria to be mounted between a slide and cover slip. Sometimes, morphological identification may be impaired, as in the case of severely damaged specimens, when mounting is not appropriately performed, or when morphological keys are not available [6]. Therefore, molecular biology approaches have been established for sand fly identification, targeting ribosomal (18S rRNA, internal transcribed spacer 2 (ITS2)) or mitochondrial markers (Cytochrome b, Cytochrome oxidase 1) [7–10]. Despite its demonstrated usefulness for studying taxonomy and population genetic markers of *Phlebotomus* spp. this technique remains time-consuming, technically demanding and expensive. Additionally, this method requires sequence information about the target genomic sequence prior to DNA amplifications and cannot be easily applied to the rapid identification and classification of new specimens [6]. Moreover, until now, no consensus genomic sequence had been defined for the molecular identification of *Phlebotomus* spp. leading to indirect comparison of studies analysing the same sand fly species with distinct genomic markers. Therefore, the development of an alternative approach using a unique strategy for phlebotomine identification has become indispensable.

Matrix-assisted laser desorption/ionization time of flight mass spectrometry (MALDI-TOF MS), which has revolutionised the identification of clinically relevant micro-organisms (bacteria, yeasts and filamentous fungi) thanks to its low cost, minimal sample preparation time and accuracy [11–13], has also been shown to be effective for arthropod identification in a pioneering study on aphid species [14]. Since then, this proteomic tool has been used for the taxonomic classification of other arthropod families such as ticks [15,16], mosquitoes [17–19], Tsetse (*Glossina* spp.) [20] and fleas [21]. Interestingly, MALDI-TOF MS has proven to be a relevant tool for field-caught biting midge identification [22,23], underlining the usefulness of this method for vector monitoring.

Recently, Dvorak *et al*. reported that MALDI-TOF MS could be successfully applied to the identification of Mediterranean sand fly *Leishmania* vector species [6]. In this study, solely laboratory-reared specimens from five sand fly species were used, and of them, two species from the same subgenus collected from two distinct areas were tested. They observed that MS profile changes were detectable between specimens from the same species with distinct geographical origins [6]. Nevertheless, more recently, the same team reported that the biomarker list was conserved between laboratory-reared and field-caught phlebotomine sand fly specimens of the same species [24]. The intra-species biomarker mass pattern maintenance between colony- or field-derived specimens enable both to be identified equally.

In Algeria, 24 phlebotomine sand flies species were inventoried [25,26], including two genera and seven subgenera [27]. Some of them are known to be *Leishmania* parasite vectors [27–29]. To prevent infections and the risk of transmission, it is necessary to map the risk areas in relation to the phlebotomine population vector by developing a reliable and rapid identification tool able to distinguish *Leishmania*-vectors from non *Leishmania*-vectors.

The aim of the present study was to establish a MALDI-TOF MS reference database of field-caught phlebotomine sand fly species captured from different geographical areas described as leishmaniasis-endemic in Algeria [30] and to evaluate the success of this database at identifying new specimens collected from the same areas. Moreover, different storage methods and the consequences of recent blood meal by the specimens on their identification by MALDI-TOF MS were tested.

## Methods

### Ethical considerations

Verbal informed consent was obtained from heads of households which were selected for sampling sand flies from inside houses.

### Field capture of sand flies

Phlebotomine sand flies were caught between June 2012 and October 2013 in four regions of North Algeria, Mostaganem (35°53’39” N, 0°05’25” E), Tizi-Ouzou (36°42’21” N, 4°17’13” E), Annaba (36°65’00” N, 7°58’33” E) and M’sila (35°35’13” N, 4°40’08” E), where cutaneous and visceral leishmaniasis have been reported (Table 1) [4,31]. Phlebotomine sand flies were caught using a suction light trap (Centers for Disease Control [John W. Hock Company, Gainesville, FL, USA, CDC]). Sand fly collection began at 6pm and ended at 7am the following morning, with a total of 13 hours collection per trap. All sand flies were pooled in order to obtain 30 specimens per tube. Sand flies which were dead at the time of collection were pooled and stored in 70% alcohol at room temperature. Conversely, live specimens were pooled and immediately frozen in liquid nitrogen and stored at -80°C until use. Two to four consecutive indoor night captures were performed per site and four light traps (Centers for Disease Control) were used per night to catch phlebotomines (Table 1).

**Table 1:** Details of the phlebotomine sand flies capture and study sites.

Study sites	Period	Number of night catches	Nature of shelter	Number of sand flies caught (storing mode: liquid nitrogen /alcohol)	Total no. of sand flies caught (catches per night per trap)
Mostaganem	June 2012	2	Goat pen	560 / 847	1407 (175.9)
Annaba	June 2013	3	Cattle pen	300 / 56	356 (29.7)
Tizi-Ouzou	August 2013	4	Chicken house	352 / 549	901 (56.3)
M’sila	July 2014	3	Chicken house	30 / 24	54 (4.5)
**Total**		**12**		**1242/1476**	**2718 (56.6)**

### Morphological identification of sand flies

The head and posterior third parts of the abdomen, bearing distinguishing characters for their identification (*i*.*e*., pharyngeal armature, genitalia and cibarium) were cut off using sterile micro-needles. These latter two compartments were placed into Marc-André solution [32] for 15 minutes at 37°C, prior to being mounted under a cover slip in a drop of polyvinyl alcohol buffer for morphological identification using morphological keys [32–34]

### Molecular identification of sand flies and *Leishmania* infectious status

The rest of the abdomen was used for DNA extraction using a Qiagen kit (Qiamp DNA mini kit, Hildesheim, Germany) according to the manufacturer’s instructions, after mechanical homogenisation as described previously [35]. Species-level molecular identification of the sand fly was performed by PCR sequencing of a fragment of the Cytochrome-b (Cyt-b) gene (Phleb_CytB_F and Phleb_CytB_R) as per the protocol used by Depaquit *et al*. [36]. For molecular detection of *Leishmania*-infected sand flies, Cytochrome-b (Cyt-b) gene (Leish_CytB_F and Leish_CytR) primers were used as described previously [37]. As positive control of *Leishmania* molecular detection, axenic *L*. *donovani* 1S2D, clone LdB, cultured as previously described [38,39] was used. The 28 sand flies in our homemade database were tested for the presence of *Leishmania* and all were negative. To amplify the mitochondrial DNA, both CytB PCRs were performed using the thermal profile as described previously [35]. The sequences were analysed using ChromasPro, version 1.34 (Technelysium Pty, Ltd., Tewantin, Queensland, Australia), and were compared to the GenBank database.

### Sample preparation for MALDI-TOF MS analysis

Thoraxes, wings and legs were manually homogenized in 10 μL of 70% formic acid and 10 μL of 50% acetonitrile (Fluka, Buchs, Switzerland) in 1.5 ml microtubes using pellet pestles (Fischer Scientific, Strasbourg, France).The homogenates were centrifuged at 10,000 rpm for 20 seconds, and 1 μL of the supernatant of each sample, corresponding to protein extract, was deposited onto a steel target plate (Bruker Daltonics, Wissembourg, France) in four spots for each sample as previously described [40]. Subsequently, 1 μL of matrix composed of saturated α-cyano-4-hydroxycynnamic acid (Sigma, Lyon. France), 50% acetonitrile(v/v), 2.5% trifluoroacetic acid (v/v) (Aldrich, Dorset, UK) and HPLC-grade water was directly overlaid on each spot sample on the target plate, dried for several minutes at room temperature and placed in the MALDI-TOF MS instrument for analysis (S1 File). To control loading on mass spectrum steel, matrix quality and MALDI-TOF apparatus performance, the matrix solution was loaded in duplicate onto each MALDI-TOF plate with or without a bacterial test standard (Bruker protein Calibration Standard I).

### MALDI-TOF MS parameters

Protein mass profiles were obtained using a Microflex LT MALDI-TOF mass spectrometer (Bruker Daltonics, Germany), with Flex Control software (Bruker Daltonics) using parameters previously described [16]. The spectrum profiles obtained were visualised with Flex analysis v.3.3 software and exported to ClinProTools software v.2.2 and MALDI-Biotyper v.3.0. (Bruker Daltonics, Germany) for data processing (smoothing, baseline subtraction and peak picking) and evaluation with cluster analysis.

### Spectrum analysis and reference database creation

Based on morphological identification, a subgroup of specimens, including the different sand fly species identified and validated by molecular biology, were selected and submitted to MALDI-TOF MS (Table 2). Intra-species reproducibility and inter-species specificity of MS spectra were evaluated using the ClinProTools 2.2 software (Bruker Daltonics). To create a database, reference spectra (MSP, Main Spectrum Profile) were created by combining the results of the spectra from at least five specimens per species using the automated function of the MALDI-Biotyper software v3.0. (Bruker Daltonics). MSP were created on the basis of an unbiased algorithm using peak position, intensity and frequency data. For rare species, fewer than five specimens were included in the database. Thus, the MS spectra from the 28 specimens used for clustering analysis were loaded into a MALDI-Biotyper v.3.0. (Bruker Daltonics, Germany) to create and increment our homemade database [41].

**Table 2:** Phlebotomine sand flies (Diptera: Psychodidae) species used to establish a MALDI-TOF MS reference database or to perform blind test 1^#^.

Morphological identification	Geographical origin/source	Number of specimens added to the database (gender^#^)	Number of specimens used for the blind test procedure	ID log score-values*[low-High]	Correct gender identification (%)
*P*. *(Larroussius) perniciosus*	Tizi-Ouzou, Mostaganem	8 (3M, 5F)	32 (20M,12F)	[2.073–2.760]	96.9%
*P*. *(Larroussius) perfiliewi*	Tizi-Ouzou, Annaba	6 (3M, 3F)	9 (3M,6F)	[2.356–2.581]	100%
*P*. *(Larroussius) longicuspis*	Tizi-Ouzou	6 (3M, 3F)	15 (9M,6F)	[2.305–2.649]	93.3%
*P*. *(Phlebotomus) papatasi*	M’sila	5 (3M, 2F)	18 (16M,2F)	[2.178–2.785]	94.4%
*P*. *(Paraphlebotomus) sergenti*	Mostaganem	1F	0		
*Sergentomyia minuta*	Tizi-Ouzou, Mostaganem	2 (1M, 1F)	1F	[1.907]	100%
**Total**		**28**	**75**	**[1.907–2.785]**	**96.0%**

^#^Correspond to sand fly specimens selected based on their morphological identification, subsequently submitted to MALDI-TOF and query to MS reference database.

*Range of log score values. M (male), F (female).

### MALDI-TOF MS biomarker mass set

To assign discriminating peaks between species and genders from *Larroussius* subgenus, 20 MS spectra from the *P*. *longicuspis*, *P*. *perfiliewi* and *P*. *perniciosus* specimens including both genders were imported into ClinProTools 2.2 software. The software was used to generate a peak list for each species in the 2 to 20 kDa mass range and to identify discriminating peaks among the analysed species. The parameter sets in ClinProTools 2.2 software for spectrum preparation were as follows: a resolution of 300; a noise threshold of 2.00; a maximum peak shift of 800 ppm and a match to calibrating agent peaks of 10%. For peak calculation, peak peaking was performed on individual spectra with a signal-to-noise threshold of 2.00 and an aggregation of 800 ppm. The spectra were then analysed using the genetic algorithm (GA) model, which displayed a list of discriminating peaks. The maximal number of peaks in model was set to 30, the maximal number of generations was set to 250 and the number of neighbours was three for KNN classification. Manual inspection and validation of the selected peaks by the operator gave a ‘recognition capability’ (RC) value together with the highest ‘cross-validation’ (CV) value. The presence or absence of all discriminating peak masses generated by the GA model was controlled by comparing the average spectra from each species.

### Blind tests for study validation

The reference spectra of each species were evaluated using two successive blind tests, with new specimens obtained from the four study sites. The level of identification significance was determined using the log score values (LSVs) given by the MALDI-Biotyper software v.3.3, corresponding to a matched degree of signal intensities of mass spectra of the query and the reference spectra. LSVs ranged from 0 to 3. Two blind tests were performed using two groups of specimens whose details are indicated below.

Blind test 1: a total of 80 non-engorged specimens stored at -80°C and morphologically identified were included in this group. Resulting MS spectra were queried against our homemade database as previously described [41], upgraded with selected sand fly specimens for database creation. Some specimens were randomly selected and subjected to molecular biology to confirm MS identification results. Moreover, to decipher incoherent results obtained between morphological and MS identification, molecular identification was performed for the respective specimens.

Blind test 2: a total of 292 specimens were included in this second test. None were initially morphologically or genetically identified. Twenty-one specimens were stored in alcohol and 271 specimens were stored at -80°C. Among the specimens stored at -80°C, 17 engorged specimens were tested separately from non-engorged ones. Approximately ten percent of the non-engorged specimens were subjected to molecular biology to verify MS identification results.

### Cluster analysis

Cluster analysis (MSP dendrogram) was performed based on the comparison of the main spectra given by MALDI-Biotyper software and clustered according to protein mass profile (*i*.*e*., their mass signals and intensities). Several clustering analyses were performed to determine how organisms are related to one another. The clustering used MSPs selected from specimens of each sand fly species. MSPs were created with MALDI Biotyper software using the default parameter for the ‘Bio Typer MSP Creation Standard Method’. To summarise, the maximum mass error of each single spectrum was 2000 Da, the desired mass error for the MSP was 200 Da, the desired peak frequency minimum was 25% and the maximum desired peak number for the MSP was 70. MSP for each specimen was created corresponding to the combination of the results of the spectra from the quadruplicate loaded on the target plate using the automated function of the MALDI-Biotyper software v3.0. (Bruker Daltonics). The 28 MSPs selected for reference MS database creation, were then used for clustering. The setting parameters were as follow: distance measure by correlation, linkage by average, the score threshold value for a single organism was 300 (arbitrary unit) and for related organisms was 0 (arbitrary unit).

### Statistical analyses

Frequencies were compared using the chi-squared test (Pearson’s chi-squared test). All differences were considered significant at *p* < 0.05 and statistical analyses were performed using the R computing environment (R Development Core Team, 2012). For multiple tests, a Bonferroni correction was applied.

## Results

### Entomological data

A total of 2,718 phlebotomine sand flies were captured. 1,476 were dead and were preserved in alcohol, while 1,242 were alive and stored at -80°C (Table 1). Of those stored at -80°C, 108 sand flies (25 from Mostaganem, 11 from Annaba, 49 from Tizi-Ouzou and 23 from M’sila) were subjected to morphological identification. Six distinct species belonging to two genera and four subgenera were identified including *P*. *(Larroussius) perniciosus* (n = 45, 26 males (M), 19 females (F)), *P*. *(Larroussius) perfiliewi* (n = 17, 7 M, 10 F), *P*. *(Larroussius) longicuspis* (n = 22, 13 M, 9 F), *P*. *(Phlebotomus) papatasi* (n = 20, 16 M, 4 F), *P*. *(Paraphlebotomus) sergenti* (n = 1, 1 F), *Sergentomyia (Sergentomyia) minuta* (n = 3, 1 M, 2 F). Some criteria used for their identification are presented in S2 File.

### Molecular identification of selected sand fly specimens and *Leishmania* infection status

Based on morphological identification, 28 specimens, encompassing the six species indexed, selected for MALDI-TOF MS database creation, were subjected to DNA extraction for molecular analyses using the remaining abdomen part (Table 2). Sequencing of the sand flies' CytB region corroborated morphological identification in all cases, and the absence of *Leishmania* parasites was also confirmed in all 28 specimens tested by molecular biology.

### MALDI-TOF MS analysis and database creation

Thorax, leg and wing supernatants of homogenates from the 28 sand fly specimens identified morphologically and confirmed by molecular biology, were subjected to MALDI-TOF MS analyses, for assessing intra-species reproducibility and inter-species specificity of MS spectra (Table 2).

**Fig 1:**
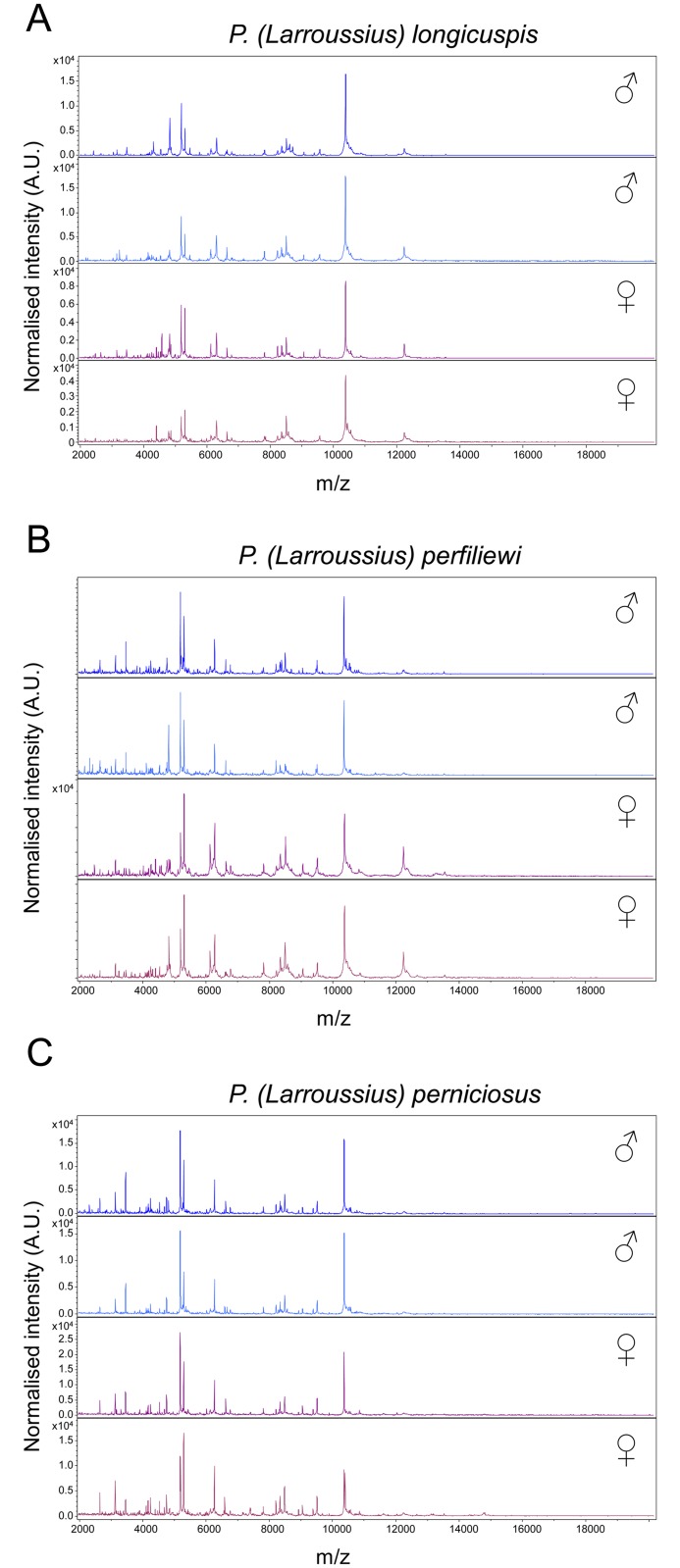
MALDI-TOF MS spectra from thorax, legs and wings protein extraction of both genders from three sand fly species. Representative MS spectra of *P*. *(Larroussius) longicuspis* (A), *P*. *(Larroussius) perfiliewi* (B), *P*. *(Larroussius) perniciosus* (C). a.u., arbitrary units; m/z, mass-to-charge ratio; ♂, male; ♀, female.

**Fig 2:**
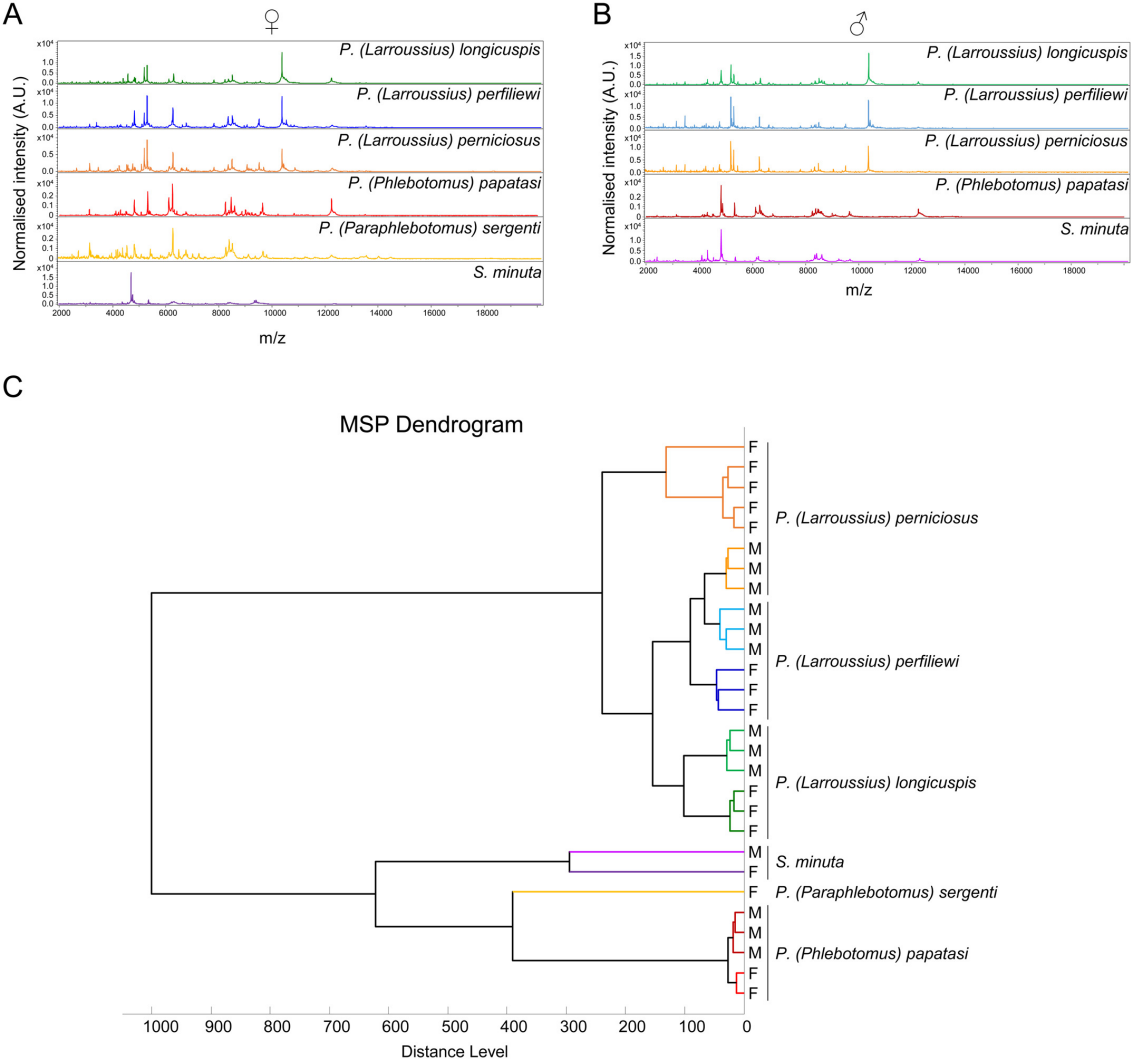
Comparison of MALDI-TOF MS spectra from thorax, leg and wing protein extraction of the six sand fly species ranging from 2 to 20 kDa. Representative spectra of females (A) and male (B) specimens are shown. The sand fly species are indicated in the right corner of each protein profile spectrum. (C) Dendrogram constructed with the 28 specimens selected for database creation (Table 2). The dendrogram was calculated by Biotyper 3.0 software and distance units correspond to the relative similarity of MS spectra. Color code is the same for each sand fly species.

Representative spectra of two specimens per gender from the three *Larroussius* subgenus are presented in Fig 1A, 1B and 1C, respectively for *P*. *longicuspis*, *P*. *perfiliewi* and *P*. *perniciosus*. Visualisation of MS profiles from all *Phlebotomus* species using Flex analysis software showed consistent and reproducible spectra for each species, moreover some MS peaks were exclusively found according to gender (Fig 1D). Although several mass peaks were shared between specimens from the same subgenus (*i*.*e*., *Larroussius*) (Fig 2A and 2B), the accurate comparison revealed distinct MS protein profiles between species indicating species-specific protein signatures. Cluster analysis based on the resulting MS profiles from the 28 selected specimens indicated clustering on distinct branches of the specimens according to species and gender (Fig 2C), confirming the reproducibility of protein profiles between specimens from the same species with gender distinction.

The Genetic Algorithm model from ClinProTools exhibited a total of 26 discriminating mass peaks, yielding recognition capability of 100% and cross validation value of 98.15% between these three species and revealing mass peaks distinguishing these species on the gender level (S3 File). Protein profiles were then specific for different species of all genera and subgenera tested with several species- and gender-unique peaks that allowed reliable and conclusive species classification of the analysed phlebotomines.

### Blind assessments of MS reference spectra database

Blind test 1: the 80 morphologically identified specimens stored at -80°C were tested against the upgraded homemade reference database. None of these specimens were found to be blood-engorged. For all the samples tested, the blind test against the database revealed log score values (LSVs) which were greater than 1.907 (Tables 2 and 3). MALDI-TOF MS identification, compared with morphological identification, revealed consistent results for more than 94% (n = 75) of the samples tested (Table 2). Of these 75 specimens, 17 (23%) were randomly selected and the sequencing of the sand fly CytB gene confirmed the accuracy of MALDI-TOF MS identification. In addition, sex determination was correct for 96% (n = 72) of the specimens tested, regardless of species. Concerning the five discordant species identifications (Table 3), the sand fly CytB gene was sequenced. Molecular biology results confirmed species identity determined by MS database questioning for these five specimens. Collectively, a threshold of LSVs greater than 1.9 was determined as relevant identification.

**Table 3:** Molecular determination from discordant results between morphological and mass spectrometry sand fly species identification.

Morphological identification	Geographical origin/source	MALDI-TOF MS identification	ID log score-values	Molecular identification (% identity)
*P*. *(Larroussius) perfiliewi*	Annaba	*P*. *(Larroussius) perniciosus*	[2.551]	*P*. *(Larroussius) perniciosus* (100%)
*P*. *(Larroussius) perfiliewi*	Tizi-Ouzou	*P*. *(Larroussius) perniciosus*	[2.211]	*P*. *(Larroussius) perniciosus* (100%)
*P*. *(Larroussius) longicuspis*	Mostaganem	*P*. *(Larroussius) perniciosus*	[2.276]	*P*. *(Larroussius) perniciosus* (100%)
*P*. *(Larroussius) perniciosus*	Mostaganem	*P*. *(Larroussius) longicuspis*	[2.191]	*P*. *(Larroussius) longicuspis* (100%)
*P*. *(Larroussius) perniciosus*	Tizi-Ouzou	*P*. *(Larroussius) longicuspis*	[2.421]	*P*. *(Larroussius) longicuspis* (100%)

### Impact of preservation mode and blood-engorged status on MS identification

**Fig 3:**
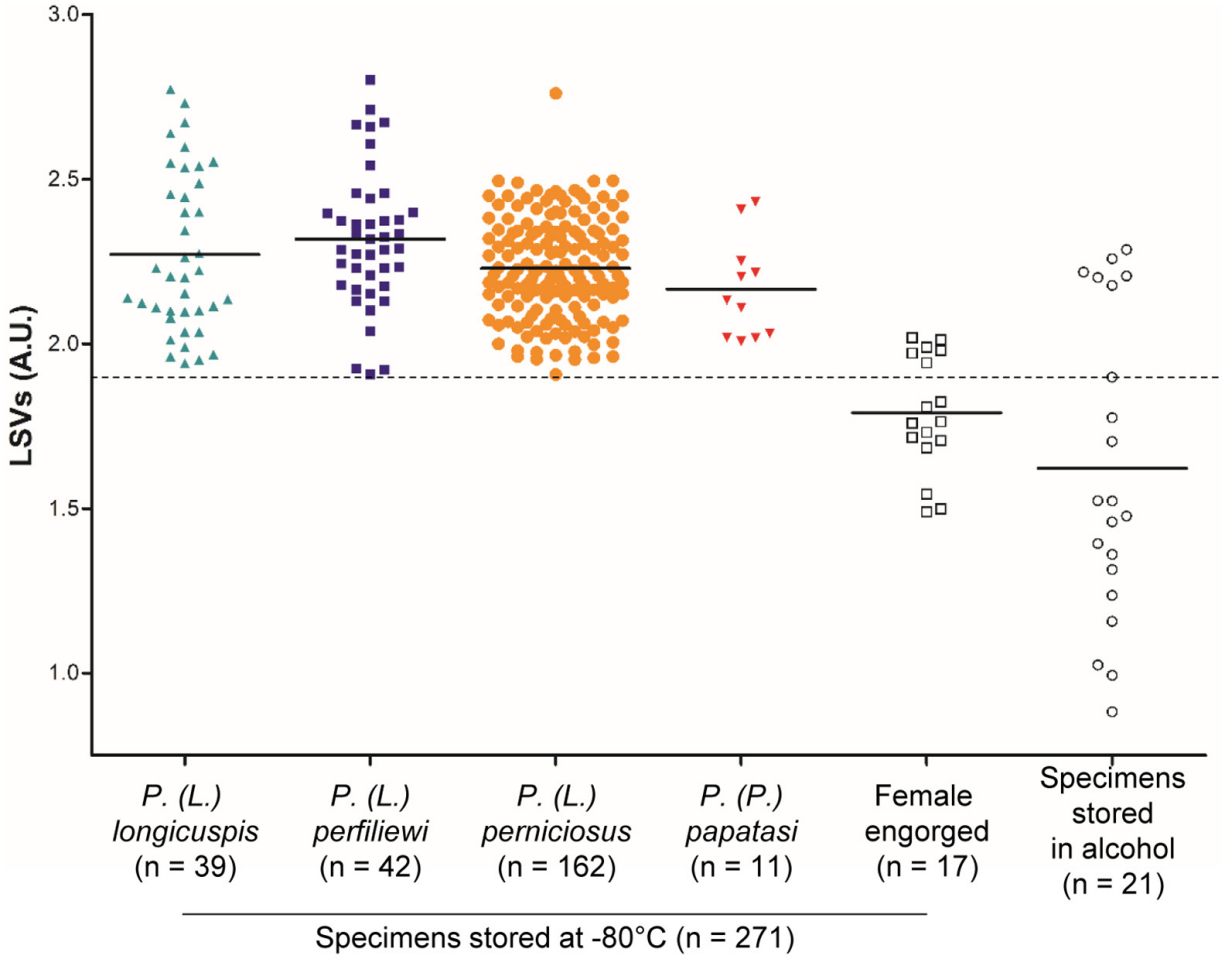
Comparison of LSVs obtained following reference arthropod database query with MS spectra of sand fly specimens according to mode of storage and engorgement status. Species-level classification is indicated only for non-engorged specimens stored at -80°C. The dashed line represents the threshold value for relevant identification (LSVs > 1.9). LSV, log score value; a.u., arbitrary units.

Blind test 2: the database query with the 254 non-engorged specimens (ratio males/females: 1.78) stored at -80°C, revealed LSVs ranging from 1.908 to 2.802 considered as relevant identification and more than 94% (n = 239) were correctly identified at the gender level (Fig 3). To control the accuracy of MS identifications, 18 specimens were blindly selected and submitted to sand fly CytB sequencing. Molecular biology results confirmed MALDI-TOF MS identification for all the samples tested.

Querying the database with the 17 engorged specimens stored at -80°C led to LSVs ranging from 0.883 to 2.286. The six engorged specimens presenting relevant LSVs (*i*.*e*., greater than 1.9), were subjected to sand fly CytB sequencing, confirming the correctness of MS identification. In terms of the 21 non-engorged specimens preserved in alcohol, LSVs ranging from 1.490 to 2.019 were obtained after reference spectrum database querying. For the six specimens with LSVs greater than 1.9, molecular biology confirmed the species identification resulting from the database query.

### Distribution of phlebotomine sand fly species between the four Algerian study sites

A total of 362 sand flies were considered for classification according to species and location, taking into account specimens used for database creation (n = 28) plus the frozen and non-engorged specimens from the first (n = 80) and second (n = 254) blind tests, (Table 4).

**Table 4:** Details of the phlebotomine sand flies fauna per study site identified by MALDI-TOF MS.

Study sites	Number of sand flies captured stored at -80°C	Number of non-engorged sand flies stored at -80°C submitted to MALDI-TOF MS (%)^(a)^	Number of *P*. *(Larroussius) perniciosus* (%)^(b)^	Number of *P*. *(Larroussius) perfiliewi* (%)^(b)^	Number of *P*. *(Larroussius) longicuspis* (%)^(b)^	Number of *P*. *(Phlebotomus) papatasi* (%)^(b)^	Number of *P*. *(Paraphlebotomus) sergenti* (%)^(b)^	Number of *S*. *(Sergentomyia) minuta* (%)^(b)^
Mostaganem	560	162 (28.9%)	115 (71.0%)	18 (11.1%)	24 (14.8%)	3 (1.9%)	1 (0.6%)	1 (0.6%)
Annaba	300	81 (27.0%)	56 (69.1%)	23 (28.4%)	2 (2.5%)	0 (0.0%)	0 (0.0%)	0 (0.0%)
Tizi-Ouzou	352	89 (25%)	34 (38.2%)	16 (18.0%)	36 (40.4%)	1 (1.1%)	0 (0.0%)	2 (2.2%)
M’sila	30	30 (100%)	0 (0.0%)	0 (0.0%)	0 (0.0%)	30 (100%)	0 (0.0%)	0 (0.0%)
**Total (%)**^(c)^	**1242**	**362 (29.1%)**	205 (56.6%)	57 (15.7%)	62 (17.1%)	34 (9.4%)	1(0.3%)	3 (0.8%)
ID log score-values*[low-High]			[1.907–2.761]	[1.908–2.802]	[1.941–2.774]	[2.008–2.785]		[1.907]

^(a)^Corresponding to the 28 specimens included in the reference database plus the 80 and 254 non-engorged specimens and stored by freezing subjected to MALDI-TOF MS analysis from blind tests 1 and 2, respectively.

^(b)^Proportion of sand fly specimens per species according to location.

^(c)^Proportion of sand fly specimens per species regardless of location.

*Range of log score values.

The 362 phlebotomine sand fly specimens represented more than one quarter of the total captured number of non-engorged and frozen specimens captured (29.1%). The three species of the subgenus *Larroussius* were predominant (89.5% of specimens identified), and were found at all study sites, with the exception of M’sila. In M’sila, all the specimens submitted to MALDI-TOF MS were identified as *P*. *(Phlebotomus) papatasi*. Interestingly, the proportion of the *Larroussius* subgenus species found in Mostaganem, Annaba and Tizi-Ouzou, were not equivalent (*p* < 1.10^−5^, chi-squared test). Pairwise comparisons of *Larroussius* subgenus species between Mostaganem, Tizi-Ouzou and Annaba indicated that *P*. *perniciosus* was found more frequently in Mostaganem than in Tizi Ouzou (*p* < 1.10^−5^, chi-squared test), while *P*. *perfiliewi* was significantly more abundant in Annaba than in Mostaganem (*p* < 0.03, chi-squared test). The frequency of *P*. *longicuspis* differed between the three pairwise comparisons (Mostaganem vs. Annaba, *p* < 0.02; Mostaganem vs. Tizi Ouzou, *p* < 0.002; Tizi Ouzou vs. Annaba, *p* < 1.10^−6^, chi-squared test). No significant differences were noted for other pairwise comparisons. Sporadic captures of *S*. *minuta* and *P*. *sergenti* were recorded.

## Discussion

Recently, MALDI-TOF MS has been successfully used for arthropod identification, including identification of laboratory-reared phlebotomine species [6]. A more recent study reported using MALDI-TOF MS for identifying counterpart sand fly species collected in the field [24]. Specimens were obtained from different countries and phlebotomine species were selected according to their *Leishmania* vectorial role [24]. However, in the field, distinct phlebotomine species could live in sympatry, some species could be vectors and others, non-vectors of *Leishmania* spp. To confirm that MALDI-TOF MS could be useful for species identification of field-caught specimens, phlebotomine sand flies caught inside animal shelters from four locations in Algeria, where human and canine leishmaniasis cases have been reported [31,42] were tested in the present study.

In line with previous studies [6,24], intra-species reproducible and inter-species specific MS patterns were obtained by MALDI-TOF MS submission of phlebotomine sand fly thoraxes, including legs and wings, independently of the collection site. Additionally, the accurate comparison of aligned MS profiles highlighted unique mass peaks allowing specimens to be classified both at the species- and gender-levels. Of the MS spectra from sand fly specimens in blind test 1 which were subjected to MALDI-TOF MS and queried against the homemade database, 94% obtained concordant identification with morphological results. The 6% of inconsistent results were attributed to morphological identification mistakes, validated by molecular biology, confirming MALDI-TOF MS identification. Finally, 100% and 96% of correct species- and gender-level identification resulted from querying of phlebotomine MS spectra from blind test 1 against the homemade database. This homemade database includes reference spectra from more than 50 arthropod species [41,43], underlining the high accuracy of MALDI-TOF MS for arthropod identification. Despite the reproducibility of MS spectra according to sand fly species and gender, males of *P*. *perniciosus* were not clustered with females of the same species. This singular clustering was supported by the genetic algorithm (GA) model displaying a list of discriminating peaks concordant with MSP dendrogram classification. Thus, the present work supports that MALDI-TOF MS was not a relevant tool for specimen classification according to phylogeny as it was repeatedly previously evoked [15, 40].

The trustworthiness of MALDI-TOF MS identification was confirmed in the second blind test for frozen non-engorged specimens giving all relevant LSVs (*i*.*e*., greater than 1.9). The lower LSV obtained for the *S*. *minuta* specimen could be attributed to several factors such as the low number of specimens queried but also the low number of *S*. *minuta* specimens included in the database, this finding extrapolated from real field specimens used for our experimentation with the abundance of some species compared to other. The threshold LSV of 1.9 was determined based on the correctness of the identification obtained from blind test 1 and 2. Moreover, 100% of the four engorged specimens reaching this threshold value were correctly identified, as confirmed by molecular biology. Collectively, based on the present results, it was rational to define a LSV cut-off of 1.9 for relevant identification. Previous studies reported that for mosquitoes [40] and ticks [16], LSVs of 1.8 were sufficient for correct identification.

A random selection of phlebotomine specimens for molecular validation corroborated the MALDI-TOF results, in contrast to morphological results. Thus, despite entomological expertise, morphological misidentification may occur. The hazy morphological identification was attributed to imperfect mounting and/or inappropriate discoloration. To decrease the risk of incorrect morphological species determination, distinguishing morphological characters of each slide-mounted specimen should be examined blindly by two entomologists. However, there are few entomologists, entomological expertise is not widespread and this method presents the great disadvantage of being time-consuming [6]. Thus, MALDI-TOF MS appears to be a relevant and competitive method for rapid and reliable phlebotomine identification, also for species from the same subgenus. MALDI-TOF MS has repeatedly been reported as a powerful tool for distinguishing closely related arthropod species such as cryptic species of *Anopheles gambiae* complex [17,19]. In the near future, the implementation of the MALDI-TOF MS reference database with spectra from specimens of other phlebotomine species will be helpful to comfort the used of this proteomic tool for sand fly identification.

Interestingly, it has been reported that the blood-engorgement of hematophagous arthropod specimens could alter MS spectra by the addition of mass peaks corresponding to vertebrate host blood [6,17]. Moreover, these additional mass peaks could differ within an arthropod species due to trophic preference attributed to gut contents [22]. To circumvent this deleterious effect, abdomens were generally dissected and excluded from the MS analysis [16,21]. However, despite the phlebotomine abdomen cut-off in the present study prior to MS analyses, only 35% of the engorged specimens which were stored frozen reach the LSV threshold of 1.9 considered as being correctly identified and validated by molecular tools. Optical microscopic analysis of engorged phlebotomine sand flies revealed traces of blood meal in the thorax. These traces of vertebrate blood could have a considerable impact on MS patterns, reducing the intensity of species-specific biomarker masses. Thus, fresh engorgement of phlebotomine sand flies appears to compromise species identification by MALDI-TOF MS. To confirm that traces of blood meal in the sand fly thorax are responsible for MS profile alteration, complementary studies evaluating new engorged field-caught female phlebotomine sand flies should be tested. To avoid this problem, other arthropod body parts could be tested. Hoppenheit A, *et al*. [20] reported that protein extracted from the wings of *Glossina* sp. was sufficient to obtain species-specific MS spectra usable for their identification. In the same way, the legs of ticks [16] or mosquitoes [19,40] have also been demonstrated as being sufficient for reliable identification at the species level by MALDI-TOF MS. The use of these body parts could effectively be an alternative strategy for circumventing the problem of accurate identification of blood-engorged sand flies based on thorax protein extract which could contain traces of mammalian blood. However, sand fly legs and wings are highly breakable and these body parts were missing on several specimens, rendering this strategy inappropriate. In order to standardise the MS spectra database for future exchanges, the same body part used by Dvorak V *et al*. [6] for sand fly identification was used.

Under field conditions, the storage of samples in 70% ethanol remains a common method of preservation. Here, the comparison of sand fly storage methods indicated that dry-freezing in liquid nitrogen liquid, followed by storage at -80°C gave the best identification results in addition to specimens preserved in alcohol. These results were in keeping with those recently published by Mathis *et al*. using MALDI-TOF MS for phlebotomine species identification [24]. Impaired arthropod identification reliability has already been reported for other arthropod species stored in ethanol [21,44]. Moreover, it is well-known that storing protein samples in ethanol for long periods of time induces protein precipitation, reducing protein solubility and, consequently, leading to a decrease in qualitative and quantitative spectra profiles, notably for masses above 9 kDa [24,45]. Thus, the present study confirmed that MALDI-TOF MS was a relevant tool for the identification of field-caught phlebotomine sand flies species, using frozen thoraxes, with some limitations for engorged females and specimens preserved in alcohol. However, supplying liquid nitrogen to secluded sites could be problematic for sand fly preservation. Other, less restrictive storage methods such as dry preservation in perforated plastic vials and storage in plastic containers with silica gel for transport to the laboratories could be an alternative [46].

Significant variations in phlebotomine sand fly density were observed among the four study sites. Higher phlebotomine specimen density was noted in Mostaganem followed by Tizou-Ouzou and Annaba, while the lowest was recorded in M’sila. Numerous factors have been shown to influence phlebotomine sand fly capture [47], notably weather conditions. Hygrometry, humidity and other environmental parameters have a considerable impact on field phlebotomine captures [47].

In the present study, nearly 30% of the specimens which were caught and frozen were submitted to MALDI-TOF MS for identification. Of the 24 recently described in Algeria [25,26], six sand fly species were found at the four prospected sites. These phlebotomine species were described as *Leishmania* vectors [4,48]. *P*. *perniciosus*, *P*. *longicuspis*, and *P*. *perfiliewi* were implicated in VL [49], whereas *P*. *papatasi* and *P*. *sergenti* were involved in CL transmission in Algeria [28]. The *Larroussius* subgenus was largely dominant in the three northern coastal sites, which represents the arthropod’s natural biotope and distribution in Algeria. Conversely, in M’sila, *P*. *(Phlebotomus) papatasi* was the only sand fly species identified at this site. Our subgenus distribution findings are comparable to previous entomological studies and phlebotomine surveys conducted in Algeria [27,28,49]. *P*. *perniciosus*, the main vector of *L*. *infantum* in Algeria [31], was found at the three coastal sites (*i*.*e*., Mostaganem, Annaba and Tizi Ouzou), although it was the dominant species at Mostaganem. This sand fly species is known to be very abundant in humid and sub-humid regions such as the coastal regions of Italy [50]. *P*. *longicuspis* displayed slight dominance in Tizi-Ouzou where this species is a proven vector of *L*. *infantum* in the same area (*i*.*e*.,Tizi-Ouzou) in Algeria [49]. *P*. *perfiliewi*, which is also a *L*. *infantum* vector, was more abundant in Annaba than in Mostaganem and Tizi-Ouzou. In M’sila, the highlands of Algeria offer a natural semiarid biotope where *P*. *papatasi* was the only species captured. *P*. *papatasi* was also the dominant species captured during an entomological survey of phlebotomine sand flies and control of CL in M’sila [42]. This species is known as a vector of *L*. *major* in Algeria [28]. Similarly to previous observations on the attraction of *P*. *perfiliewi* and *P*. *perniciosus* to large animals [51], these two species were found in goat and cattle shelters. Although *P*. *papatasi* is known to be highly endophilic and anthropophilic and observed in domestic resting sites in urban areas, its presence in chicken houses is becoming very common in many areas of Italy and Tunisia [51,52].

In Algeria, *P*. *perniciosus* and *P*. *longicuspis* are widely distributed and sympatric in some foci, especially in the northern and central parts of the country [27]. Recently, Pesson *et al* [53] and Boudabous *et al* [54] in Morocco and Tunisia, respectively, reported a widely distribution of atypical morphs of *P*. *perniciosus* presenting single-pointed aedeagi curved at their apicies that were indistinguishable from the *P*. *longicuspis* aedeagi. However, in eastern Algeria, the atypical morphs of *P*. *perniciosus* were until now never reported [55]. Here, these results were supported by the morphological analyses of aedeagi and Cytb sequencing from *P*. *perniciosus* and *P*. *longicuspis* specimens, revealing the presence of uniquely typical morphs *P*. *perniciosus* as previously described in Tunisia [54]. Further studies with a representative number of specimens from different Algerian geographical areas could elucidate the distribution of typical and atypical forms of this sand fly species.

Collectively, our data support previous findings concerning the distribution of phlebotomine species at the geographical, density and environmental levels. Despite the fact that none of the specimens tested was infected by *Leishmania* parasites, the different proportions of three *Larroussius* subgenus species, living in sympatry, suggest that VL vectors could be distinguished according to site.

### Conclusions

The present work has confirmed that MALDI-TOF MS may represent a rapid and inexpensive alternative tool for accurate identification of phlebotomine sand fly species collected in the field. Differences in phlebotomine species density in the study sites confirmed that VL vectors are predominant in at-risk coastal sites, while the only sand fly species vector of CL was recorded in the highlands. The recent demonstration of the use of MALDI-TOF MS for the accurate detection of *Rickettsia* spp. [41] and *Borrelia crocidurae* [56] in ticks, offers new investigative avenues, such as the opportunity to determine, in a single analysis, the identity of phlebotomine sand flies at the species level and their *Leishmania*-infectious status. The development of such tools for *Leishmania* detection in phlebotomine sand flies will revolutionise epidemiological surveys of this vector.

## Supporting Information

S1 File Details of specimens included in each test. (DOCX)

S2 File Photographs of decisive morphological criteria for identification of the six sand fly species inventoried. Sand fly spermathecae (B, D, F, J, L and M) and male genitalia (A, C, E, G and H) are presented. Pharyngeal teeth (head) of *P*. *(Phlebotomus) papatasi* (F), (magnification x40) is indicated in panel K. Male (A) and female (B) *P*. *(Larroussius) longicuspis*, (magnification x40); Male (C) and female (D) *P*. *(Larroussius) perniciosus*, (x40); Male (E) and female (F) *P*. *(Larroussius) perfiliewi* (x40); (G) (x40) and (I) (x20): Enlarged sections of Male (H) *P*. *(Phlebotomus) papatasi* (x5); Female (J and K) *P*. *(Phlebotomus) papatasi* (x40); Female (L) *Sergentomyia* (*Sergentomyia) minuta* (x40); Female (M) *P*. *(Paraphlebotomus) sergenti* (x40). Spermathecae basis are indicated by hashtags (#), spermathecae ducts by arrowheads (►) and spermathecae by asterisks (*).
(TIF)

S3 File Mass peaks list discriminating the three sand fly species and gender from (*Larroussius)* subgenus. (DOCX)
