# Supplementary material for: Identification of Algerian Field-Caught Phlebotomine Sand Fly Vectors by MALDI-TOF MS
Source: PLoS Negl Trop Dis. 2016 Jan 15;10(1):e0004351. doi: 10.1371/journal.pntd.0004351 (PMC4714931; doi:10.1371/journal.pntd.0004351)
Supplement: S1 File — (DOCX) [file pntd.0004351.s001.docx]

**Additional file S1.** Details of specimens included in each tests

| **Group tests** | **Number of specimens morphologically identified** | **Number of specimens submitted to MS identification** | **Number of specimens subjected to molecular identification** | **Number of specimens per engorged status** | **Number of specimens per storage method** |
| --- | --- | --- | --- | --- | --- |
| Database creation | 28 | 28 | 28 | 28 / non engorged | 28 /-80°C |
| Blind test 1 | 80 | 80 | 17 | 80 / non engorged | 80 /-80°C |
| Blind test 2 | 0 | 292 | 18 | 275/ non engorged  17 / engorged | 271 /-80°C  21 / alcohol |
