# Supplementary figures and images for: Identification of Algerian Field-Caught Phlebotomine Sand Fly Vectors by MALDI-TOF MS
Source: PLoS Negl Trop Dis. 2016 Jan 15;10(1):e0004351. doi: 10.1371/journal.pntd.0004351 (PMC4714931; doi:10.1371/journal.pntd.0004351)

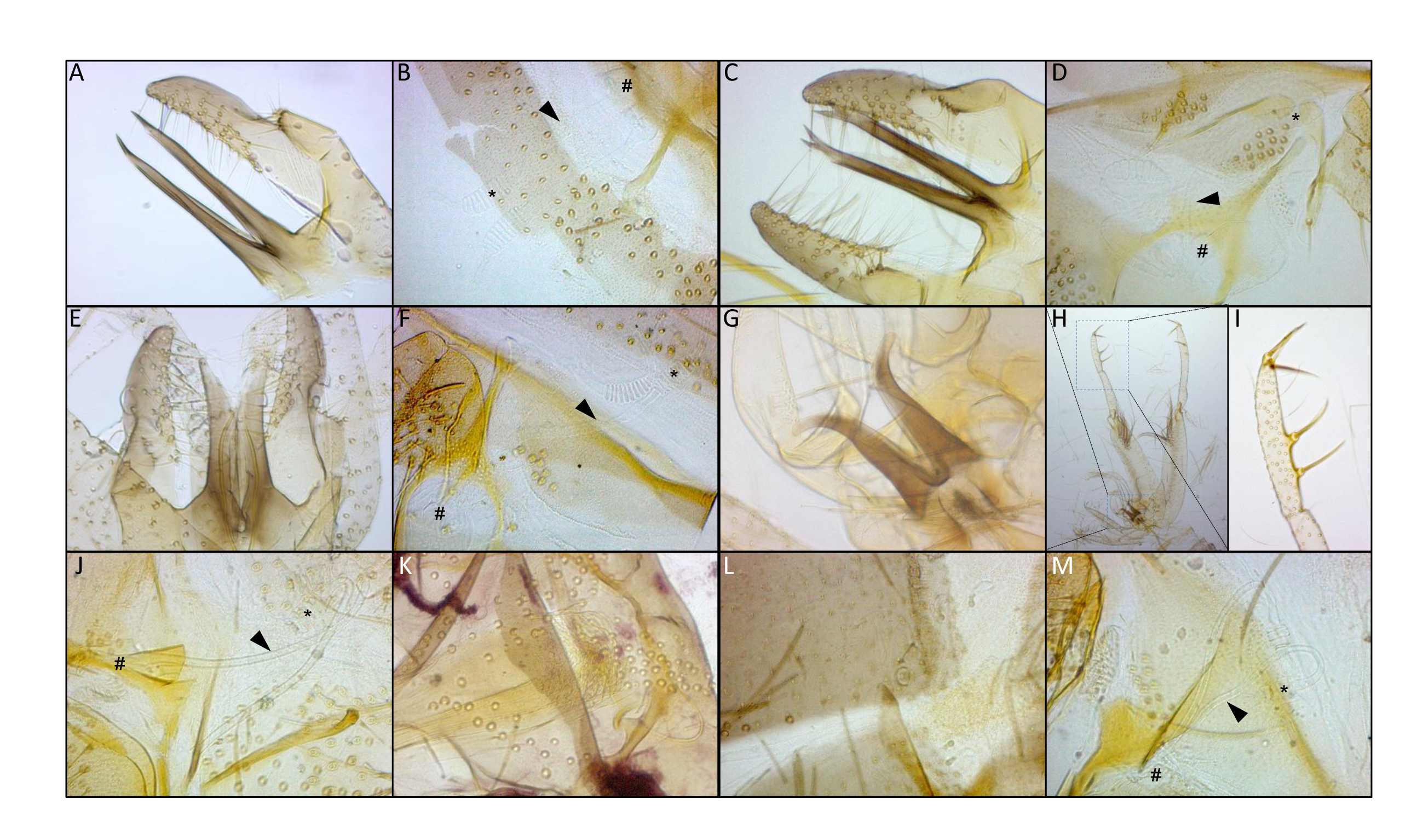

Supplement: S2 File — Sand fly spermathecae (B, D, F, J, L and M) and male genitalia (A, C, E, G and H) are presented. Pharyngeal teeth (head) of P. (Phlebotomus) papatasi (F), (magnification x40) is indicated in panel K. Male (A) and female (B) P. (Larroussius) longicuspis, (magnification x40); Male (C) and female (D) P. (Larroussius) perniciosus, (x40); Male (E) and female (F) P. (Larroussius) perfiliewi (x40); (G) (x40) and (I) (x20): Enlarged sections of Male (H) P. (Phlebotomus) papatasi (x5); Female (J and K) P. (Phlebotomus) papatasi (x40); Female (L) Sergentomyia (Sergentomyia) minuta (x40); Female (M) P. (Paraphlebotomus) sergenti (x40). Spermathecae basis are indicated by hashtags (#), spermathecae ducts by arrowheads (►) and spermathecae by asterisks (*). (TIF) [file pntd.0004351.s002.tif]
