# Supplementary material for: Identification of Algerian Field-Caught Phlebotomine Sand Fly Vectors by MALDI-TOF MS
Source: PLoS Negl Trop Dis. 2016 Jan 15;10(1):e0004351. doi: 10.1371/journal.pntd.0004351 (PMC4714931; doi:10.1371/journal.pntd.0004351)
Supplement: S3 File — (DOCX) [file pntd.0004351.s003.docx]

**Additional file S3.** Mass peaks list discriminating the three sand fly species and gender from (*Larroussius)* subgenus

| Number | Mass m/z [Da]* | Start Mass m/z [Da] | End Mass m/z [Da] | P. (Larroussius) longicuspis | | P. (Larroussius) perfiliewi | | P. (Larroussius) perniciosus | |
| --- | --- | --- | --- | --- | --- | --- | --- | --- | --- |
|  |  |  |  | Female | Male | Female | Male | Female | Male |
| 1 | 3136.73 | 3123.34 | 3150.67 | - | - | + | + | + | + |
| 2 | 3459.56 | 3446.22 | 3461.02 | + | + | + | + | - | + |
| 3 | 4252.22 | 4234.86 | 4254.59 | - | - | - | - | + | + |
| 4 | 4257.09 | 4254.59 | 4273.94 | + | + | + | + | - | - |
| 5 | 4306.18 | 4296.88 | 4323.79 | + | + | + | + | - | - |
| 6 | 4857.66 | 4856.56 | 4880.63 | + | - | - | - | - | - |
| 7 | 5364.37 | 5361.52 | 5392.73 | - | - | - | - | + | - |
| 8 | 6272.13 | 6250.66 | 6289.91 | - | - | + | + | + | + |
| 9 | 6302.49 | 6289.91 | 6324.54 | + | + | - | - | - | - |
| 10 | 6336.63 | 6324.54 | 6365.81 | - | - | - | - | + | + |
| 11 | 6399.04 | 6383 | 6425.48 | - | - | - | - | + | - |
| 12 | 7812.56 | 7794.71 | 7821.35 | - | - | - | + | - | + |
| 13 | 7821.35 | 7821.35 | 7854.48 | + | + | + | - | + | - |
| 14 | 7887.22 | 7863.77 | 7919.75 | - | - | - | - | + | - |
| 15 | 8214.93 | 8189.95 | 8223.11 | - | - | - | + | - | + |
| 16 | 8228.35 | 8223.11 | 8228.35 | - | - | + | - | + | - |
| 17 | 8235.75 | 8228.35 | 8261.4 | + | + | - | - | - | - |
| 18 | 8500.7 | 8473.23 | 8508.29 | - | - | - | - | + | + |
| 19 | 8508.92 | 8508.29 | 8515.04 | + | + | + | - | - | - |
| 20 | 9515.06 | 9485.09 | 9522.2 | - | - | - | + | - | + |
| 21 | 9524.96 | 9522.2 | 9547.17 | - | - | + | - | + | - |
| 22 | 9568.66 | 9547.17 | 9584.08 | + | + | - | - | - | - |
| 23 | 10365.36 | 10343.71 | 10377.2 | - | - | - | + | - | + |
| 24 | 10377.2 | 10377.2 | 10410.98 | + | + | + | - | + | - |
| 25 | 10445.09 | 10419.05 | 10458.53 | - | - | - | - | + | - |
| 26 | 12252.19 | 12211.52 | 12253.66 | + | + | + | - | - | - |
| Total |  |  |  | 11 | 10 | 11 | 9 | 13 | **10** |

*Used of specimens included in the reference MS database and ClinProTool software for mass peak list determination.
